# Surprising Prokaryotic and Eukaryotic Diversity, Community Structure and Biogeography of Ethiopian Soda Lakes

**DOI:** 10.1371/journal.pone.0072577

**Published:** 2013-08-30

**Authors:** Anders Lanzén, Addis Simachew, Amare Gessesse, Dominika Chmolowska, Inge Jonassen, Lise Øvreås

**Affiliations:** 1 Department of Biology and Centre for Geobiology, University of Bergen, Bergen, Norway; 2 Computational Biology Unit, Uni Computing, Uni Research AS, Bergen, Norway; 3 Institute of Biotechnology, Addis Ababa University, Addis Ababa, Ethiopia; 4 Institute of Environmental Sciences, Jagiellonian University, Krakow, Poland; 5 Department of Informatics, University of Bergen, Bergen, Norway; University of Glasgow, United Kingdom

## Abstract

Soda lakes are intriguing ecosystems harboring extremely productive microbial communities in spite of their extreme environmental conditions. This makes them valuable model systems for studying the connection between community structure and abiotic parameters such as pH and salinity. For the first time, we apply high-throughput sequencing to accurately estimate phylogenetic richness and composition in five soda lakes, located in the Ethiopian Rift Valley. The lakes were selected for their contrasting pH, salinities and stratification and several depths or spatial positions were covered in each lake. DNA was extracted and analyzed from all lakes at various depths and RNA extracted from two of the lakes, analyzed using both amplicon- and shotgun sequencing. We reveal a surprisingly high biodiversity in all of the studied lakes, similar to that of freshwater lakes. Interestingly, diversity appeared uncorrelated or positively correlated to pH and salinity, with the most “extreme” lakes showing the highest richness. Together, pH, dissolved oxygen, sodium- and potassium concentration explained approximately 30% of the compositional variation between samples. A diversity of prokaryotic and eukaryotic taxa could be identified, including several putatively involved in carbon-, sulfur- or nitrogen cycling. Key processes like methane oxidation, ammonia oxidation and ‘nitrifier denitrification’ were also confirmed by mRNA transcript analyses.

## Introduction

Soda lakes are strongly alkaline lakes, typically with a pH between 9 to 11, high concentrations of carbonate ions and with salinities ranging from brackish to hypersaline [1]. Although relatively rare, these lakes constitute a large part of inland water by volume in certain regions, particularly arid or semi-arid areas connected to tectonic rifts such as the East African Rift Valley. In spite of their basicity, many soda lakes show unusually high primary productivity, including the highest photosynthesis rates measured in any aquatic habitat (above 7 g C m^−2^ day^−1^) [2]. Thus, they rank not only as the most productive but also among the most extreme aquatic ecosystems. Not all soda lakes are highly productive, however, and the mechanisms controlling primary production may involve many factors such as nutrient limitations, toxicity, or trophic interactions [3]. Salinity, however, may be the strongest stress factor limiting microbial diversity [4], [5]. In spite of this, high morphological diversity comparable to neutral freshwater systems has been observed, even in hypersaline soda lakes [3].

In this study, we address the diversity of these fascinating ecosystems, challenging the notion that extreme habitats generally harbor lower biodiversity. Further, we investigate whether a relationship between salinity and taxonomic richness exists in the investigated soda lakes. The underlying question we attempt to answer is to what extent salinity, pH and other parameters influence the microbial community structure. We also address whether lakes located closer together generally harbored more similar communities.

A number of alkaliphilic microorganisms with key metabolic roles in soda lakes have previously been studied using both cultivation dependent and independent techniques. Such studies have uncovered a diversity of various functional and phylogenetic groups including cyanobacteria (e.g. [6]), anoxygenic phototrophs (e.g. [7], [8], [9], [10]), aerobic heterotrophs (e.g. [10]), sulfur reducers and other anaerobic organotrophs (e.g. [3]), sulfur oxidizers (reviewed in [11]), acetogens (e.g. [5]), methanogens (e.g. [12]), methylotrophs (e.g. [13], [14], [15]) or eukaryotic microorganisms (e.g. [16]). Others addressed planktonic community composition and diversity of individual lakes [17], [18] or across several lakes, including soda lakes [19], [20], by using molecular fingerprinting or SSU rRNA clone libraries. However, such techniques are limited in terms of their resolution or ability to determine the phylogenetic diversity and community structure at high resolution [21].

This study is the first to use high throughput sequencing to analyze the structure of soda lake microbial plankton communities. Using a combination of traditional marker gene profiling and PCR-independent shotgun sequencing of reverse transcribed rRNA, we target both the active (RNA) and present (DNA) diversity and composition in these intriguing ecosystems. This approach has been shown to provide a more holistic view [22], [23], enabling quantitative analysis of taxonomic groups from all domains of life simultaneously as well as a snapshot of abundant mRNA transcripts.

## Results

### Characteristics of Investigated Lakes

Five soda lakes were selected based on contrasting characteristics such as salinity, size and location. Lakes Abijata, Chitu and Shalla are located more centrally within the East African Rift and share higher salinities compared to Beseka and Arenguadi, located in the Upper Awash Basin and Central Ethiopian Highlands, respectively. While Arenguadi and Chitu are relatively small crater lakes (the former a maar), Shalla is the largest studied and represent the only deep lake, with maximum and average depths of 266 and 87 m, respectively [24]. While Abijata is retracting due to water diversion and soda ash extraction [25], Beseka is expanding [26], resulting in changing salinities and phytoplankton communities [27]. Tables 1 and S1 list the sampling sites, collected samples and physicochemical parameters measured.

**Table 1:** Overview of the soda lakes, samples and sequence datasets studied.

		*Sampling*			*Physical parameters*					*Number of datasets*			
Lake		Spots^a^	Depths	Area (km^2^)	pH	Na (ppm)	K (ppm)	Salinity (%)	Oxy-cline	DNA amplicon	cDNA amplicon	Prefilter^b^ amplicon	cDNA shotgun
Abijata		3	1 (0 m)	176	9.9	11,460	457	3.4	–	3	0	0	0
Arenguadi		1	5 (0–30 m)	0.54	9.7–9.9	1,254	227	0.21–0.28	3.5 m	5	5	0	1
Beseka		1	3 (0–13 m)	44	9.6	1,605	60	0.29–0.31	–	3	3	1	1
Chitu		3	3 (0–15 m)	0.8	10.4	18,430	1,136	5.8	<0.5 m	6	0	1	0
Shalla		1	3 (0–30 m)	329	9.8	7,623	253	1.8	–	3	0	0	0
	***Total*** *:*									*20*	*8*	*2*	*2*

a GPS coordinates measurements for individual depths and other details are listed in Table S1.

b DNA Amplicon library prepared from 5 µm “pre-filter” at 0 m depth.

Significant stratification was only encountered in Lake Arenguadi, saturated in oxygen until a depth of 3 m, followed by a narrow oxycline and then anoxia below 4 m. Subtle changes in salinity and pH were also encountered between limnia (Table S1). The holomictic lake Chitu appeared recently mixed during sampling. Only low levels of oxygen were measured at water surface until about 10 cm depth with no discernible salinity or pH gradients.

### Composition and Diversity of the Microbial Communities

In total 458,813 sequence reads representing SSU rRNA were obtained from DNA amplicon- (n = 22), cDNA amplicon- (n = 8) and shotgun sequence (n = 2) datasets, in addition to 6,745 putative mRNA reads (Tables 1 and S2). The “prefilter” samples from lakes Beseka and Chitu, yielded disproportionately large and small numbers of sequence reads, respectively.

**Figure 1:**
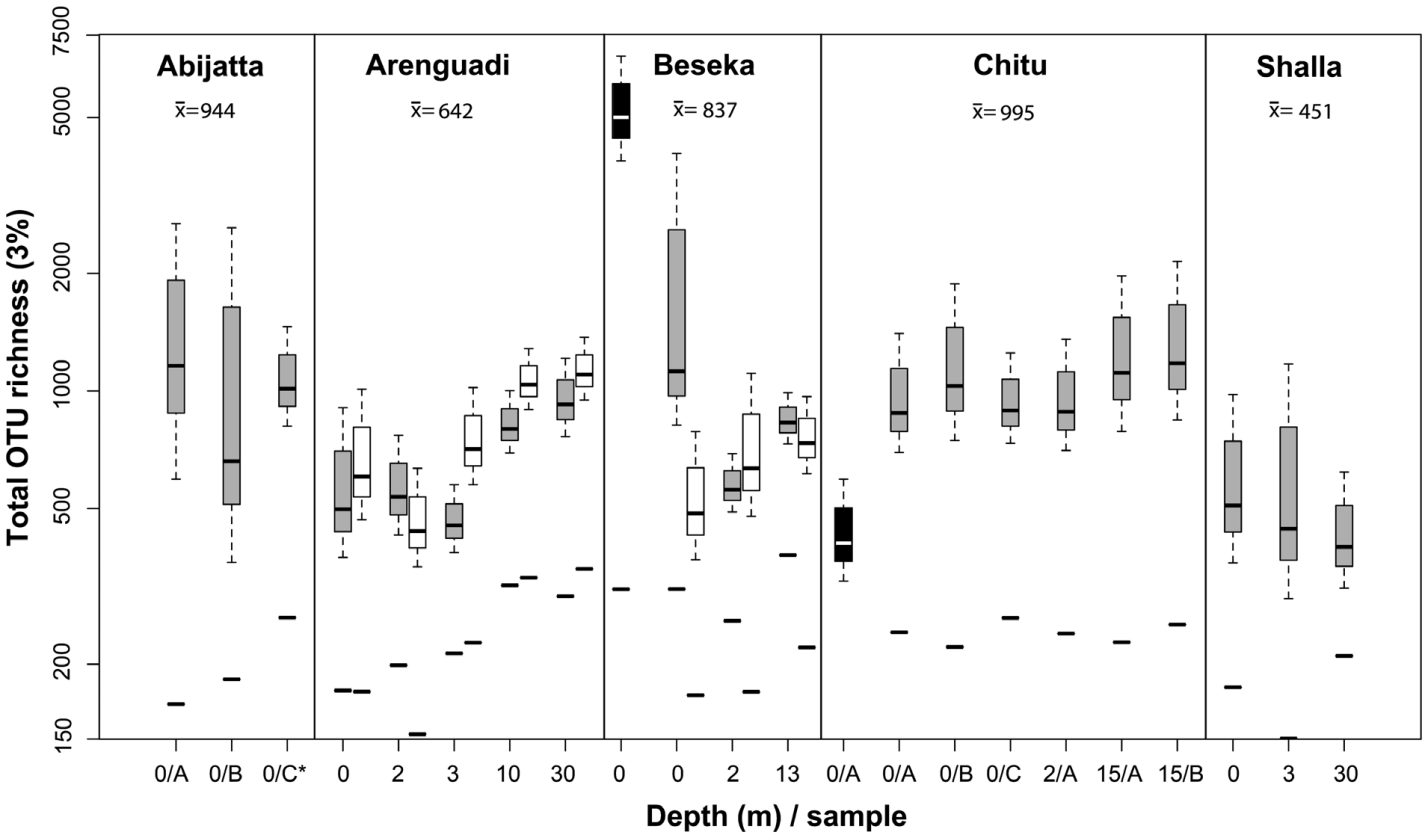
Parametric richness estimates. Box-plots cover 95% Bayesian confidence intervals of total OTU richness for each sample. Grey boxes indicate DNA amplicon datasets, white boxes cDNA amplicons and black boxes DNA amplicon datasets derived from prefilters. Solid lines below the box plots indicate rarified OTU richness. Arithmetic means of medians for DNA amplicon datasets (excluding prefilter-derived) are shown below lake names.

Total OTU richness amounted to 2,704 (3% distance), excluding 1,286 singletons. OTUs per dataset varied between 169 and 1,519 (Table S2). As indicated by rarefaction analysis (Fig. S1), sequencing depth was far from being exhaustive even in the largest dataset. Estimated Shannon diversity (*H*′) varied between 2.3 and 4.7, showing no correlation to the number of reads, as opposed to OTU richness (Table S2). However, the substantial variance of *H′* between spatial replicates inside of Abijata and Chitu was similar to variance between lakes, indicating that differences in *H’* between lakes were not significant, at least lacking better replication. Instead, Bayesian parametric estimation of total richness [28] was used to compare diversity between datasets in a more accurate manner. The Sichel distribution fit best to the observed prokaryotic OTU-abundance distributions in most datasets (28 of 30) and was used to calculate confidence intervals of total sample richness, illustrated in Figure 1. Medians of estimated richness ranges generally followed the same trend as rarefied OTU richness, but the later varied more across spatial replicates, consistent with the variance of *H′*.

The highest median richness was estimated in the surface of Beseka and the lowest at 30 m depth in Shalla. Significantly higher richness (α = 0.05) was estimated from several datasets compared to the later. From means of estimated richness in DNA datasets (excluding prefilters; Fig. 1), Chitu appears to be the most diverse lake, closely followed by Abijata. The RNA-derived datasets showed similar richness estimates as their corresponding DNA datasets and followed the same trend, except in two cases (Fig. 1). Firstly, estimated RNA richness was significantly lower in the Beseka surface sample. Secondly, in Arenguadi at 2 m, significantly higher richness was predicted in RNA. Predicted richness in the stratified Lake Arenguadi followed a trend remarkably similar to that of cell density, as estimated using DAPI staining (Fig. S2).

### Comparisons of Community Structure and Influence of Physicochemical Parameters

**Figure 2:**
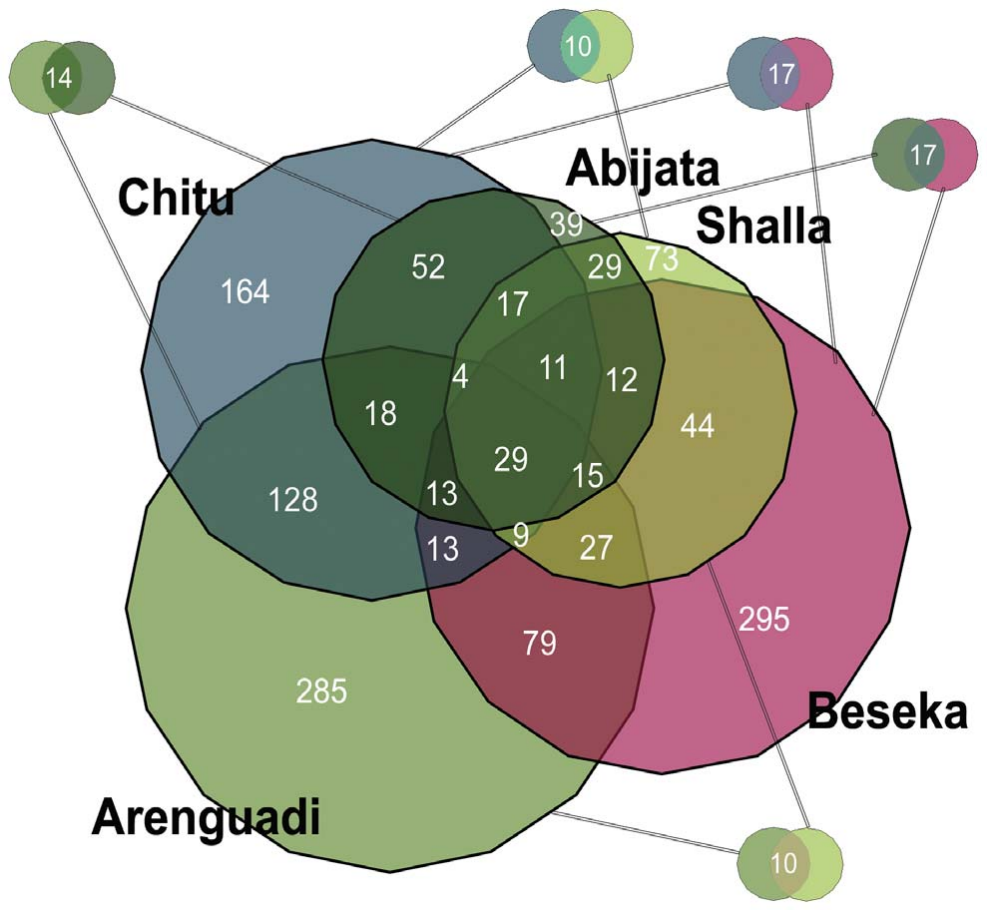
Venn diagram showing the distribution of shared OTUs across lakes. White numbers indicate the number of OTUs in each possible subset, adjusted for differences in sequencing depth.

Figure 2 shows the distribution of OTUs across lakes (excluding prefilter- and cDNA-derived plus adjusted for contrasting sequencing depths). Abijata and Shalla showed a relatively larger overlap than other lakes, while Beseka harbored most OTUs unique to one lake. Larger proportions of OTUs were shared between different depths in the same lake, compared to those shared between lakes, particularly for RNA-derived datasets (Fig. S3).

**Figure 3:**
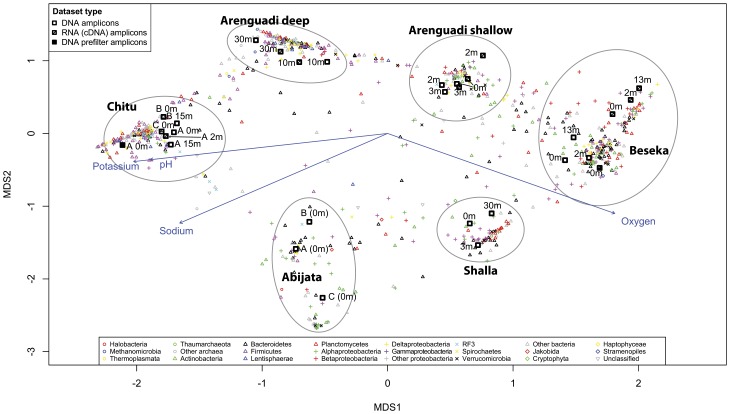
Non-metric multidimensional scaling (NMDS) based on Bray-Curtis dissimilarities between OTU compositions of individual datasets. Sequence datasets OTUs and fitted physicochemical parameters are plotted on the first two NMDS axes. The colors and shapes of individual OTUs and sequence datasets represent their taxonomical classification or dataset type, according to the legends.

Hierarchical clustering (Fig. S4) and Non-metric multidimensional scaling (NMDS; Fig. 3) based on OTU composition (Bray-Curtis dissimilarities) showed that all datasets formed lake-specific clusters, except for Arenguadi where the anoxic hypolimnion (10 and 30 m, “Arenguadi deep”) formed a separate cluster from the oxic epilimnion (0–3 m, “Arenguadi shallow”). The former appeared more similar to Chitu, representing the other anoxic environment; and the later to Beseka, representing the other low-salinity lake. The same clustering pattern was obtained using taxonomical distributions rather than OTUs, with the two shotgun-sequenced datasets clustering with their respective lakes (Fig. S5). Based on the observed clustering pattern, six “habitat” clusters were defined.

As indicated by NMDS, community samples within lakes were more similar than those from different lakes, with the exception of the two layers found in Arenguadi. This pattern was confirmed comparing dissimilarities (Bray-Curtis) between shallow samples inside the same lakes (for Arenguadi and Chitu), to those between different lakes (using average compositions for replicate samples). According to a Welch t-test the difference in similarity was significant (p<10^−15^).

In order to evaluate the influence of lake water composition and other measured physicochemical parameters, a separate NMDS was constructed from pooled habitat datasets. Out of the parameters, four showed significant correlation to this NMDS: dissolved oxygen (presence or absence), pH, sodium- (Na^+^) and potassium (K^+^) concentrations. These parameters also correlated significantly to the NMDS made from un-merged datasets and their fitted vectors have been added in Fig. 3. Variation partioning analysis suggested that taken together, these parameters explained 29% of the variation in community composition between habitats and 31% between individual datasets (Fig. S6). The influence of distance between lakes on community dissimilarity was also investigated using linear regression (Fig. S7). A weak correlation may exist, but was not significant among the lakes studied. Comparisons between Chitu and nearby lakes formed obvious outliers.

### Most Abundant Taxa and mRNA Transcripts

**Figure 4:**
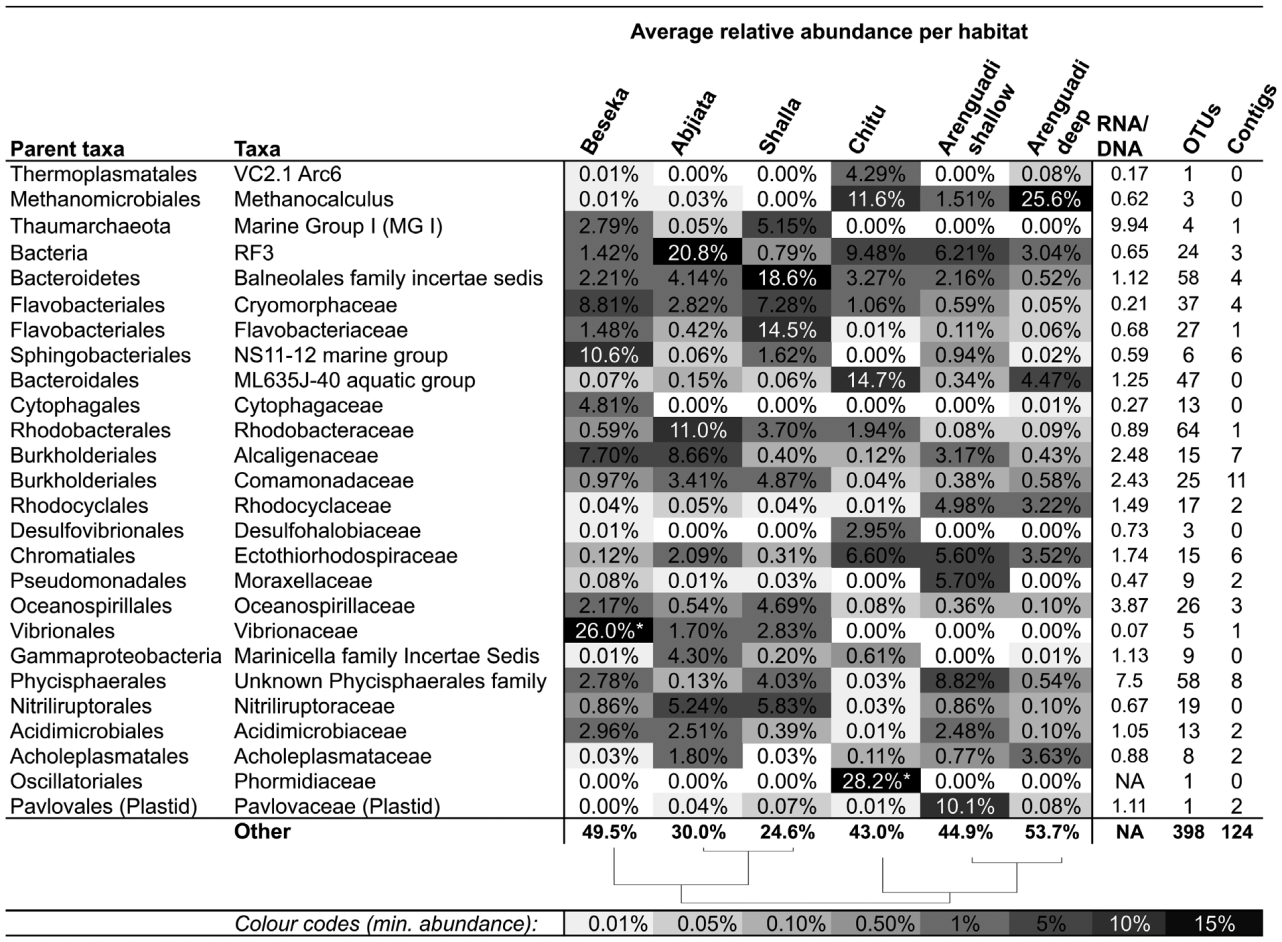
Distribution matrix with, DNA/RNA ratio, number of OTUs and rRNA contigs for the five most abundant taxa in each habitat. Abundances are based on DNA amplicons from collection filters except those indicated with a star (*), instead based on prefilter-derived datasets. Taxa were defined at family level except for RF3 and MG I where information was not available at this resolution. DNA/RNA ratios are based on the dataset with highest RNA abundance and number of rRNA contigs include only those >750 bp. The dendogram indicate average linkage clustering of habitats based on OTU distribution (BC-dissimilarity).

Figure 4 shows the distribution across habitats of abundant taxa at family rank or below, based on amplicon sequencing (prokaryotes and plastids only). It also lists RNA/DNA abundance ratios indicating the relative metabolic activity, number of OTUs and rRNA contigs. Together these cover 46–75% of total reads retrieved from each habitat. Table S3 lists the complete taxonomical composition for each rank and dataset. Few taxa were abundant in all six habitats, the deep-branching RF3 being an exception (min. abundance 0.8%). This phylum-level clade includes uncultured sequences from soda lakes, deep-sea sediments and enterosymbionts, with similarity to the prokaryotic genus incertae sedis *Gemella* [29]. *Methanocalculus* had the second highest average abundance, while Marine Group 1 *Thaumarchaeota* had the highest RNA/DNA-ratio and *Rhodobacteraceae* the highest diversity with 64 OTUs.

Table 2 lists all environmental datasets containing sequences most similar to amplicon or rRNA contigs from the abundant taxa included in Figure 4. This included datasets from seven alkaline lakes, eight saline or brackish-, and six non-saline bodies of water with unknown or neutral pH. It also included three datasets from soil and two cultured isolates: *Rhodobaca bogoriensis* [8] and a symbiont of *Hydra magnipapillata* [30]. Sequences derived from Mono Lake, California were the most commonly encountered amongst those most similar to abundant taxa.

**Table 2:** Sequences from environmental samples and cultured isolates similar to abundant taxonomic groups.

Habitat	Description	Region	Reference	Similarity to taxa*
Mono Lake	Meriomictic and saline soda lake.Water and sediments sampled.	California	[17] and AF448167–AF448198**	RF3 (99*%), Balneolales,* ML635J-40 aquatic group (*Bacteroidales)*, *Thioalkalivibrio*, *Marinicella* (100%), *Nitriliruptoraceae* (99%), *Acidimicrobiaceae*
Lonar Lake	Meriomictic and saline crater sodalake. Sediments sampled.	India	[77] and JQ738919–JQ739136**	ML635J-40 aquatic group (99%), *Rhodobacteraceae* (99%), *Rhodocyclaceae*, *Thioalkalivibrio*, *Oceanospirillaceae*
Soap Lake	Meriomictic and saline soda lake.	Washington State, USA	[7], [18]	RF3
Kulunda Steppe lake	S-reducing, plus methano-genicsoda lake isolates	Altai, Russia	[78] and JQ837890–5**	*Desulfohalobiaceae* (99%), *Methanocalculus*
Xiarinur Lake	Sediment samples from salinesoda lake.	Inner Mongolia	GU083676–88, GQ848203–9**	*Thioalkalivibrio*
Qinghai Lake	Brackish soda lake	Tibet	HM127307–HM127858* *	RF3, *Balneolales*, *Oceanospirillaceae*, *Acidimicrobiaceae*
Mahoney Lake	Stratified lake with alkaline epilimnion	British Columbia	[39]	RF3 (99%)
Lake Bonney	Permanently ice-covered saline lake	Antarctica	[79]	*Balneolales*
Lake Zabuye	Hypersaline soda lake	Tibet	[80]	*Cryomorphaceae*, *Rhodobacteraceae* (99%)
Salton sea	Moderately alkaline, hypersaline lake.	California	[81]	*Flavobacteriaceae*
Salt marsh sediments	Archaeal clone library from Barn Island tidal marshes	Connecticut	[82]	VC2.1 Arc6 (*Thermoplasmatales*)
Coastal water	Beaufort Inlet	N Carolina	JN233293**	*Cryomorphaceae*
Hypersaline biofilm	Hypersaline microbial mat	Guerrere Negro	[83]	*Thioalkalivibrio*
Chesapeake Bay	Brackish estuary	NE USA	[84]	*Acidimicrobiaceae*
Dongping Lake	Freshwater lake	China	FJ612110– FJ612447**	*Cryomorphaceae* (99%), NS11-12 marine group (99%)
Anderson Lake	Shallow freshwater lake(part of Warner Lakes)	Oregon	EU283511**	NS11-12 marine group,
Lake Kauhako	Meromictic, moderatelysaline crater lake	Hawaii	AY344367– AY344440**	*Rhodobacteraceae* (99%), *Phycisphaerales*
Wuliangsuhai Lake	Shallow freshwater lake	Inner Mongolia	FJ820362–FJ820488**	Alcaligenaceae (99%), *Comamonadaceae* (99%)
Contaminated groundwater	High levels of nitric acid-bearinguranium waste	USA	AY661997**	*Moraxellaceae* (99%)
Hydrothermal vent	Deep-sea vent chimneys	Juan de FucaRidge	EU559823**	VC2.1 Arc6 (*Thermoplasmatales*)
Gold mine 1	Geothermal water	Japan	[56]	MG I *Thaumarchaea* (99%)
Gold mine 2	Soil from mine shaft	USA	[55]	MG I *Thaumarchaea* (100%)
Saline soil	Saline, coastal soil	India	[85]	*Cytophagaceae*
Contaminated soil	Petroleum-contaminated alkalineand saline soil	China	JF421131**	*Oceanospirillaceae*
Swamp/lab strain	Putative symbiont of *Hydra magnipapillata*	Japan	[30]	*Acholeplasmataceae*
Lake Bogoria	Isolate from soda lake	Kenya	[8]	*Rhodobacteraceae* (99%)

* Abundant taxa from this study for which highest-scoring alignments match sequences from the environmental dataset or isolate. Similarity given in brackets when above 98%.

** Accession numbers to rRNA sequences without published manuscripts.

Our complementary cDNA shotgun sequencing approach allowed insights into the most abundant eukaryotic taxa in lakes Beseka and Arenguadi (Table 3). The primers used also amplified chloroplast rRNA for all photosynthetic eukaryotes encountered, in some cases improving the insight into their distributions. For example, the most abundant eukaryotic family encountered in Arenguadi, *Pavlovaceae*, appeared more abundant at 2 m than at the surface of the lake and was also encountered in Chitu.

**Table 3:** Ten most abundant eukaryotic taxa.

Taxon	Arenguadi*	Beseka*	Other lakes*
*Pavlovaceae*	4.83% (19.4%)	0.00%	Shalla: 0.2%, Chitu: 0.1%, Abijata: 0.1%
*Geminigeraceae*	0.33% (0.19%)	2.74% (3.32%)	Shalla: 1.6%, Abijata: 0.4%
*Chroomonadaceae*	0.00%	2.71% (1.14%)	–
*Chlorophyceae* **	0.01%	2.33% (1.17%)	–
*Thalassiosiraceae*	0.00%	0.14% (4.20%)	–
*Placididea* **	0.15%	0.51%	–
*Bicosoecidae*	0.76%	0.01%	–
*Dysteriida* **	2.80%	0.00%	–
*Cyclidiidae*	0.76%	0.01%	–
*Didiniidae*	0.45%	0.11%	–

* Highest relative abundance out of lake-specific datasets given (sample from 2 m for *Pavlovaceae* and prefilter at 0 m for *Thalassiosiraceae*).

** Classification beyond this taxonomic rank uncertain.

Further, diatoms from the family *Thalassiosiraceae* showing low abundance in the shotgun dataset were the most abundant eukaryotic taxon in the prefilter-derived dataset. Beseka appeared to harbor a contrasting eukaryotic community compared to Arenguadi, with phototrophs instead dominated by the mentioned diatoms, cryptophytes and *Chlorophyceae.* Non-phototrophic flagellates were present in both datasets, but with *Placididea* dominating in Beseka and *Bicosoecidae* in Arenguadi. Ciliates from different families were present in both lakes but more abundant in Arenguadi with *Dysteriida* constituting 2.8% of the sequences (see Table 3).

UniRef protein sequence clusters matching abundant putative mRNA-transcripts are listed in Table S4. Transcripts of Photosystems I and II were the most abundant of the genes with known functions (n = 39 in Arenguadi, n = 49 in Beseka). Various flagellar gene transcripts were also abundant in both lakes (n = {29, 12}). In addition, Arenguadi contained many transcripts from particulate methane monooxygenase (n = 26), others similar to a gene involved in calcium binding (A0L9Q4; n = 10), Chaperone DnaK (n = 7), and viral genes such as RNA-directed DNA polymerase (n = 10), RNA-directed RNA polymerase (n = 5), capsid and structural proteins (n = 4). Beseka instead contained transcripts from ammonia monooxygenase (*amoA*, n = 11) and nitrite reductase (n*irK*, n = 4).

### Effect of Filtering

It is possible that the pre-filtering of water samples biased the community structure in collected biomass. Most OTUs encountered from collection-filters of the surface samples from Beseka were also encountered from the prefilters (Fig. S3), but often at contrasting abundances. In order to assess this “prefilter-bias”, taxon abundances were compared between the datasets derived from prefilters and corresponding collection-filters. A comparison was also made between the dataset derived from centrifugation (*LAb C*) and those from collection-filters. All consistent and significant outcomes of these comparisons are presented in Table 4. Eight of ten affected taxa showed decreased abundances, i.e. were more likely to pass through the prefilter. Seven of these were also less abundant in *LAb C*, at ratios indicating a more severe bias than from pre-filtering. Two taxa showed the opposite influence, i.e. increased abundance on prefilters, both from the phylum *Planctomycetes*.

**Table 4:** Families with relative abundance consistently influenced by filtering in lakes Arenguadi and Beseka.

Name	RP_1_	Significance*	RP_2_
VC2.1 Arc6	0.02	* in Beseka	N/A
*Desulfuromonadaceae*	0.16	* in Beseka	0.2
Unknown *Sphingobacteriales* family	0.2	* in Chitu	0.03*
*Cryomorphaceae*	0.22	* in Chitu	0.1
RF3**	0.22	* in both lakes	0.01
Unknown *Flavobacteriales* family	0.3	* in Chitu	0.02*
*Ectothiorhodospiraceae*	0.31	* in both lakes	0.2
*Rhodobacteraceae*	0.59	* in Chitu	36*
Unknown *Phycisphaerales* family	2.2	* in Chitu	0.4
*Planctomycetaceae*	2.9	* in Chitu	6*

RP_1_: Average ratio of proportions for taxon abundance derived from prefilters compared to collection-filters.

RP_2_: Ratio of proportions for comparison of abundances in *LAb C* (centrifugation harvested) relative LAb A and B.

* Significant change (p<0.05, after Bonferroni correction).

** Taxonomy at family rank not available.

Several abundant taxa in prefilter-derived datasets were completely missing or uncommon in datasets from collection-filters. An example is *Arthrospira platensis* constituting 28% of the prefilter sequences in Chitu (Fig. 4, fam. *Phormidiaceae*; Table S3), indicating that most cells from these taxa could not pass the prefilters.

## Discussion

### Phylogenetic Diversity

All soda lakes studied harbored remarkably diverse microbial communities, considering their high pH. This also applied to pre-filter samples including filamentous or particle-associated organisms mostly missing from downstream collection filters. Surprisingly, the lake with the most extreme conditions (anoxic Lake Chitu) yielded the highest OTU richness, followed by the lake with the second highest pH and salinity (Abijata). The two anoxic samples from the stratified Lake Arenguadi also yielded relatively higher richness than surface samples. This is clearly a blatant transgression of the common notion that more extreme habitats should be less diverse. It even indicates the opposite: a positive correlation between phylogenetic richness and salinity or pH. To test this intriguing, counter-intuitive hypothesis properly, however, a larger number of replicates and lakes would be needed, evenly distributed along salinity and pH gradients.

In addition, cell density appeared correlated with diversity across depths in Arenguadi and when comparing to Lake Shalla, whose cell density was one order of magnitude lower (Figures 1, S2). Although our estimates were limited to these two lakes, a previous study estimated similar values of bacterial cells per volume in Arenguadi, placing Chitu and Abijata at about half its cell density, Shalla and Beseka about one order of magnitude below [31]. This agrees with the hypothesis that pH and salinity also increases richness. The effect these parameters have on productivity and prokaryotic cell density is challenging to disentangle and may involve complex trophic interactions, as grazers are generally more sensitive to salinity and pH. Although not measured in this study, the primary productivity rate is also expected to play an important role in these interactions.

The parametric richness estimation used compensates for contrasting sequencing depth, but relies on an assumption that sequence datasets constitute a representative subsample of the underlying biological community [28]. There are several problems with this assumption, including bias introduced from sampling, rRNA gene copy number [32], nucleic acid extraction [33] and PCR [34]. While these are expected to bias all amplicon datasets in a similar manner, cell density might not. However, concentrations of extracted nucleic acid did not follow the same trend as cell densities (Table S2), Further, template concentrations were adjusted prior to PCR, to avoid such bias. Thus, the correlation between diversity and cell density was likely not artificial.

The implicit richness definition used here was OTUs per volume unit, since the same sample volume was collected from each lake and mixed before filtering. Similar sample volumes were also filtered (Table S1). With larger cell density, we thus sampled more cells, more likely to represent higher richness. Rather than a sampling bias, this is arguably a general issue with comparing richness between habitats of contrasting biomass, area or volume [35].

Although no published studies utilised cloning-free high throughput sequencing to estimate the diversity of soda lake water samples, Xiong *et al.* [36] used it to analyze lake sediments, identifying a negative correlation between pH and richness. This disagrees with our hypothesis for planktonic diversity, but it is clearly possible that benthic communities show different correlations to these factors. Studies of Tibetan lakes [37] and the Baltic Sea [38] have examined similar salinity ranges as that studied here. Both identified a strong influence of salinity on community composition, but not richness. As opposed to pH and salinity, previous findings support the finding of anoxic hypolimnia being more diverse than corresponding epilimnia [17], [39], [40]. The cause of this is equally intriguing and possibly due to a high degree of endemism [41], challenging another common notion, namely that “everything is everywhere” [42].

OTU richness in surface [43] and hypolimnion [40] samples of neutral freshwater lakes has previously been studied using the same sequencing platform and noise-filtering as employed here (AmpliconNoise) [44]. The range of rarefied OTU richness from our soda lake datasets (Fig. S1) is approximately half of that obtained in these studies (300–600 at 5,000 reads in the former and 74–392 in the later). However, these studies targeted the V3–V4 regions of SSU rRNA instead of V5–V6, possibly resulting in higher richness estimates, not comparable to ours [45], [46]. It also appears that the pre-filtering used here prevented detection of several taxa. The observation of taxonomic richness comparable to neutral freshwater lakes agrees well with previous observations of morphological [3] and molecular diversity [20].

Richness of RNA-derived datasets was comparable to that of DNA-derived datasets in most samples, indicating that the majority of diversity originated from the active community, rather than an inactive “seed bank”. Although total active richness of RNA cannot theoretically be higher than that of available DNA, richness estimates suggested this in one of the samples (Arenguadi 2 m; Fig. 1). This may be explained by PCR bias affecting RNA-derived (cDNA) template in a different manner than the relatively longer DNA template. This was supported by RNA-derived datasets from Arenguadi having significantly higher rates of detected chimeras than DNA counterparts (Table S2). Artifacts introduced during reverse transcription may also have caused it.

### Community Composition and Correlation to Physicochemical Parameters

Clustering and NMDS analyses supported both by OTU- and taxonomic composition, divide the datasets into six well-separated groups or habitats: one for each lake except Arenguadi, where epi- and hypolimnion were separated. Most of the dominant taxa show sharp abundance differences across habitats (Fig. 4) and shallow samples inside the same lake were significantly more similar to each other than those from different lakes. Likewise, relatively few OTUs were shared between more than one or two habitats (Fig. 2), compared to those shared between depths (Fig. S3) or spatial replicates. This difference was more pronounced in RNA-derived datasets. This is expected in an ecosystem where the activities of taxa (RNA) are more strongly influenced by local conditions than the DNA pool, also containing a “seed bank” of inactive and sporulating organisms and thus expected be more randomly distributed, spatially [47].

Out of OTUs shared between lakes (Fig. 2), two pairs: Abijata and Shalla; as well as Arenguadi and Chitu, showed larger overlaps between them than other lakes. The former overlap may be explained by the fact that Shalla and Abijata are located close together and were connected as recently as 2,000 years ago [48]. As for Arenguadi and Chitu, these were the only two lakes to contain samples from anoxic environments. Thus, obligate anaerobes were only shared between them out of the lakes studied. Except for Chitu, there is some support for the notion that sampled lakes located closer together harbored more similar communities than those far apart (Fig. S7). Although not significant, this could indicate distance-dependent dispersal limitations.

Out of the measured parameters, oxygen (presence or absence), pH, Na^+^ and K^+^ concentrations were significantly correlated to the OTU composition in the studied habitats. Although oxygen appeared to have the largest influence when partitioning the compositional variation in pooled habitat-datasets, Na^+^ was equally important when including individual datasets (Fig. S6). The relative contributions of pH and K^+^ were equally hard to disentangle. Regardless of model used, these parameters explain about 30% of community variation. As mentioned, salinity and oxygen have previously been established as important factors for shaping the microbial composition in aquatic habitats [16], [38], [41]. Na^+^ and pH have also been indicated as important influences for OTU composition in soda lake sediments [36].

### Taxa Encountered and Possible Ecological Roles

We expect that the amplicon datasets obtained were representative for the majority of taxa in the underlying community of bacterial and archaeal plankton. This was confirmed using complementary shotgun sequencing, alternative DNA extraction- and harvesting protocols, for the pre-filter- and *LAb C* samples. Resulting datasets conformed to habitat-specific clustering patterns and shared similar abundances for most taxa, compared to corresponding amplicon datasets from default protocols. Exceptions include *Arthrospira*, *Thalassiosiraceae* and *Planctomycetes*, whose abundances were dramatically decreased by pre-filtering. This is expected, considering these taxa have filamentous growth, large rigid cells and attach to surfaces or other cells, respectively. Correspondingly, underrepresented taxa are good candidates for having smaller than average cells and it appears these were not successfully collected using centrifugation.

Due to filtering bias, it was challenging to identify the main primary producers in the lakes studied. *Arthrospira platensis* appeared to dominate the surface of Chitu. This was also the only lake with large flocks of Lesser Flamingos present during sampling. These birds are typically found together with *Arthrospira*, which is their main diet [49]. This genus was only present in trace amounts in Arenguadi, consistent with earlier reports that it is disappearing from the lake [50]. Instead, abundance of photosynthetic taxa was dominated by the Cyanobacterial genera *Leptolyngbya* and *Anabaenopsis*, but mainly by the eukaryotic haptophyte *Pavlovaceae*. The later family is a flagellated unicellular algae commonly found in brackish littoral costal waters [51]. Chloroplastic 16S from *Pavlovaceae* was also present in other lakes, but two orders of magnitude less abundant. At genus rank, most reads of this family were classified as *Pavlova,* while the only full-length 18S rRNA contig obtained from the taxon was more similar (99%) to *Diacronema* (AF106056). However, these two genera appear polyphyletic and a merger has been suggested [51].

No cyanobacteria were detected in Abijata, Shalla or Beseka, probably due to filtering bias. Given the lack of cyanobacterial reads, it is probable that photosynthesis in Beseka was dominated by eukaryotes. Compared to Arenguadi, a different and more diverse community of photosynthetic eukaryotes was present, dominated by cryptophytes in the families *Geminigeraceae* and *Chroomonadaceae*. Mostly studied as model organisms for secondary endosymbiosis, these are flagellated and unicellular, like the *Pavlovaceae.* Also abundant were *Chlorophyceae*, mainly unclassified at higher ranks, and diatoms of the family *Thalassiosiraceae*.

Anoxygenic photosynthesis also appeared to contribute to primary production in several lakes. A diversity of non-sulfur purple bacteria from the family *Rhodobaceraceae* (genera *Rhodobaca*, *Rhodobacter*, *Pseudorhodobacter* and *Roseibacter*) dominated in Abijata and Shalla, while purple sulfur bacteria from the genus *Ectothiorhodospira* dominated in the anoxic lake Chitu and also occurred in Abijata. The non-phototrophic genus *Thioalkalivibrio* in the same family (*Ectothiorhodospiraceae*) was abundant in lakes Chitu and Arenguadi. An internal sulfur cycle is suggested by the presence of sulfate reducers from the families *Desulfohalobiaceae* (mainly *Desulfonatronovibrio*), as previously observed in soda lakes [3]. Both *Thioalkalivibrio* and *Desulfonatronovibrio* are known as widespread and diverse groups commonly found in soda lakes [11]. Most similar environmental sequences from other studies were also from soda lakes. No obvious sulfate reducers could be identified in Arenguadi. It is possible that hydrothermal springs feeding some of the lakes studied contain sulfide of geological origin, although no studies supporting this could be identified.

Methanogens, mainly from the genera *Methanocalculus Methanolobus* and *Methanoseata* were found, with the highest relative abundance in Arenguadi (at 30 m) and Chitu. A single OTU classified as *Methanocalculus* dominated among these, most similar to isolates from a soda lake on the Kulunda Steppe (Table 2). The most similar (98%) validly described isolate was *M. halotolerans*, a hydrogenotrophic and methylotrophic species isolated from an oilfield [52]. Aerobic methane oxidation in the surface of Arenguadi was evident from mRNA transcripts and presence of *Methylomicrobium*, previously encountered in soda lakes [13].

Ammonia-oxidizing archaea (AOA) from Marine Group I *Thaumarchaeota* [53] were found in high abundance in lakes Shalla and Beseka, constituting as much as half of the RNA-derived reads at 13 m depth in Beseka while also abundant at the surface. Although sequences from AOA inhabiting soda lakes exist [54], those encountered here were more similar to environmental sequences from two different gold mines [55], [56] (Table 2), belonging to the terrestrial subgroup Lambda I [57]. Active ammonia oxidation was confirmed by active transcription of archaeal *amoA*, as well as *nirK*. The later observation is particularly interesting as it supports the suggested role of AOA in ‘nitrifier denitrification’ [58], recently demonstrated in soil [59], marine habitats [60] and enrichment cultures [61] including estuaries with similar salinity to Lake Beseka [62].

It is possible that *Planctomycetes* were involved in anaerobic ammonia oxidation (anammox), although none of the known anammox taxa [63] were encountered. The type species of the most common order found (*Phycisphaerales*) is instead a heterotrophic algae symbiont [64]. It is possible that nitrogen fixation is carried out by *Rhodobacter* in Lake Shalla, *Azoarcus (*fam. *Rhodocyclaceae*) in Arenguadi and *Derxia* in Beseka (as well as other taxa in fam. *Alcaligenaceae*). Putative denitrifiers include *Rhodobacteraceae*.

Other taxa encountered at high abundance include aerobic heterotrophs (e.g. *Bacteroidetes*, *Moraxellaceae*, *Marinicella*) and fermentative anaerobes (e.g. *Thermoplasmatales*). Taxa typical for highly specialized metabolisms were also encountered such as *Oceanospirillaceae* and *Nitriliiruptor,* the later known for being able to catabolize nitriles or cyanides [65]. Others, like RF3, remain poorly studied and with unknown function. Many in both categories showed high similarity to sequences found previously in saline or soda lakes (Table 2).

A diversity of putatively bacterivorous eukaryotes was present including ciliates (*Dysteriida*, *Cyclidiidae, Didiniidae* etc.), flagellates (*Bicosoecidae*, *Placididea*, *Colpodella* etc.), rotifers (*Polyarthra*, *Brachionus*), *Simocephalus, Cercozoa* and *Heterolobosea*, the most abundant listed in Table 3. Considering their abundance and diversity, it is probable that these exert a considerable top-down control on the prokaryotic community. To what extent viruses control the diversity and structure of the microbial community can only be guessed. A few putative bacteriophage transcripts were found among the limited mRNA reads from Lake Arenguadi. Transcripts from information processing genes were found in higher abundance, however, from (+)ssRNA- and retrovirus, groups known to only infect eukaryotes.

## Materials and Methods

### Sample Collection, Filtering and Storage

Sampling in Abijata-Shalla National Park was carried out with permission and supervision from the Ethiopian Wildlife Conservation Authority. No permission was required for the other two lakes (Beseka and Arenguadi), located in publicly accessible areas.

Water samples (excluding Abijata sample C; “*LAb C*”) were collected in March 2011 using a 2.5L Niskin bottle (Ocean Scientific International Ltd.), kept in sealed containers and pre-filtered using 5 µm polycarbonate filters (Poretics Ø47 mm, Osmonics Inc. USA.) in order to avoid immediate clogging of more narrow collection filters suitable for prokaryotic cells. The filtrate was then passed through 0.2 µm Sterivex™ columns (Millipore) until clogging occurred, in order to maximize cell yield. Site names, coordinates, depths and filtered volumes are listed in Table S1. While prefilters were deposited in 15 mL Falcon tubes filled with RNALater, Sterivex columns were filled with RNALater and sealed. All samples were stored at 4°C until further processing.

Sample *LAb C* was collected in December 2011 and processed using a different, more rapid protocol, mainly for evaluation purposes. Surface water was collected using sterile 50 ml Falcon tubes, transported on ice to Addis Ababa University, then preserved at 4°C for less than a week. Isohaline PBS (pH 11) was added to the sample and biomass harvested by centrifugation at 3700 RPM for 30 min at 4°C from 200 ml water by repeatedly removing supernatant and adding new water using a Consul 21R centrifuge (Orto Alresa). Finally, cell pellets were washed with PBS and centrifuged twice to remove salt particles. Pure cell pellets were preserved at −20°C until DNA extraction.

### Measurements of Physicochemical Parameters

Concentrations of Na^+^ and K^+^ and a number of other ions were measured from native surface water samples (stored at 4°C in 15 mL Falcon tubes), using inductively coupled plasma optical emission spectrometry (Elemental IRIS, Thermo Fisher Scientific Inc.). Salinity, pH and dissolved oxygen (DO) was measured on site during sampling: total salinity with a standard refractometer (0–100‰, ATAGO Co. Ltd.); pH with a portable pH-meter (Oakton pH 110, Eutech Instruments Pty. Ltd.) and confirmed with indicator strips (Merck, range 5–10); and DO using a portable dissolved oxygen meter (Hi9143, Hanna Instruments). Due to equipment failure, the oxygen level could not be measured properly in lakes Abijata, Beseka or Shalla. To compensate, DO was instead treated as a binary variable in future analysis (presence or absence), based on read-outs and earlier measurements. All sampled depths in the problematic lakes were determined as nearly saturated (presence).

### Cell Enumeration

Unfiltered water samples were collected in 15 ml Falcon tubes and filled with formaldehyde to a concentration of 2%. DAPI staining was used for enumeration of total prokaryotic cells. Formaldehyde-fixed water samples were thoroughly mixed by vortexing, 1 ml aliquots dried on 0.2 µm filters, incubated with 2% DAPI solution for 15 min in the dark, then rinsed with sterile distilled water (2×10 ml). Filters mounted on microscope slides were inspected using a Zeiss Axioplan fluorescent microscope and manually counted in diagonal squares of an overlaid grid. Mean and standard errors of cell densities were calculated using a minimum of 12 squares per sample.

### Nucleic Acid Extractions

DNA and RNA was simultaneously extracted directly from Sterivex columns using the AllPrep DNA/RNA Extraction Kit (Qiagen). Prior to extraction, columns were opened, RNALater removed and replaced with lysis buffer (RLT Plus). The columns were then re-sealed, rotated gently and incubated for 1 minute before lysate was passed through the filter by manual air pressure application using a syringe. Subsequent extraction steps were carried out according to the manufacturer’s protocol and extracts stored at −80°C. From *LAb C* and prefilters, community DNA was extracted using CTAB as described previously [66]. Extracted DNA pellets were dried and resuspended in 50 µl of TE buffer (pH 8) and stored at −20°C. Nucleic acid concentrations were determined using NanoDrop™ spectrometry.

### cDNA Synthesis

Total RNA was quality assessed using gel electrophoresis. Extracts where RNA was detected, while lacking well-contrasted bands corresponding to the two ribosomal subunits were discarded, retaining only those from Arenguadi and Beseka. From these lakes, single-strand reverse transcription was carried out to provide template for amplicon libraries. Superscript III (Invitrogen) was used according to the manufacturer’s protocol, random hexamer primed and with subsequent RNAse H digestion. In addition, the two surface samples were subjected to double-stranded cDNA synthesis as described previously [23].

### Amplicon Library Preparation

PCR amplification of the V5–V8 region of prokaryotic SSU rRNA (16S) was carried out from extracted DNA and single-stranded cDNA using the primers Uni787F (5′-ATTAGATACCCNGGTAG-3′) and Uni1492R (50-GNTACCTTGTTACGACTT-30) [67] using a two-step (nested) PCR protocol described previously [68]. Template concentrations and number of PCR cycles (Table S2) were adjusted to achieve equal concentrations of final products. Triplicate PCR reactions were pooled and purified using GenElute PCR Clean-Up kit (Sigma) prior to the second PCR step, instead using primers with attached sample-specific, error-correcting barcodes (“multiplex identifiers”) and GS-FLX adaptors (Lib-L). Resulting amplicons were cleaned using AMPure XP (Beckman Coulter) following the manufacturer’s protocol (bead-to-sample ratio 9∶10). Amplicon DNA was analyzed using gel electrophoresis to ensure complete removal of primers and negligible amounts of non-barcoded product. Concentrations were measured using Qubit and amplicons stored at −80°C until pooling in equimolar amounts and sequencing.

### Sequencing and Data Submission

Pyrosequencing, ds-cDNA synthesis and shotgun library preparation was carried out at the Norwegian High-Throughput Sequencing Centre. Amplicons were sequenced using GS-FLX Titanium chemistry (Lib-L) and cDNA shotgun libraries using GS-FLX+. No fragmentation was carried out since sequences longer than 3000 bp (DNA-contamination) were rare. Resulting flowgrams were submitted to the NCBI Sequence Read Archive with study accession number SRA061754.

### Sequencing Processing, Including Filtering and Noise-removal

In amplicon datasets, filtering, removal of noise and chimeric sequences was carried out using AmpliconNoise (AN) [44]. This method shows the most complete removal of PCR and sequencing artifacts, while not obfuscating real, OTUs [69]. Barcode and primer sequences were removed and resulting sequences annotated with read-abundance. In addition to the chimera filtering carried in AN (Perseus), UCHIME [70] was used to remove any remaining chimeric sequences (min. score 0.1) and SilvaMod106 as reference database [29]. The resulting “cleaned” sequences were clustered into OTUs using maximum linkage based on pairwise Needleman-Wunsch alignment distances at a 3% distance cutoff using AN [44]. Diversity indices (1-D and *H’*) were calculated from resulting OTUs using the *OTUDist.sh* script distributed with AN (v1.26 alpha) and rarefaction carried out using the program E-Rarefaction [28]. Rarefied richness was based on the smallest dataset, excluding the Chitu prefilter (2,967 reads). Shotgun cDNA reads were filtered by removing reads shorter than 150 bp, with degenerate bases (‘Ns’) or average quality below 25.

Cleaned amplicon sequences and filtered shotgun reads were subjected to taxonomic classification using CREST [29]. Assembly of full-length rRNA contigs was carried out independently using shotgun reads from taxonomic groups as described previously [71]. Shotgun reads with an alignment bitscore below 50 were screened and cleaned for ncRNAs using Infernal and Rfam [72], [73], then aligned to UniRef90 [74] using BLASTX to identify putative mRNA transcripts (min. bitscore 45).

### Ordination, Variation Partitioning and Other Statistical Analyses

Calculation of Bray-Curtis dissimilarities between datasets as well as hierarchical clustering, NMDS, parameter correlation and variation partitioning based on these, were carried out using the R programming language [75] and the Vegan package [76]. To support OTU-based analysis, taxonomic groups were derived from the number of reads assigned to each taxon at all ranks from domain to genus using the composite *All_Composition.txt* output from CREST [29]. Taxonomic comparison of datasets derived from prefilters vs. collection filters, as well as shotgun sequencing vs. amplicons, was carried out as described previously [22].

## Supporting Information

Figure S1 **Rarefaction curves of OTUs from amplicon samples.** The number of encountered OTUs (perceived richness) is plotted relative to sub-sampled sequence datasets size, i.e. number of reads. For Chitu and Abijata, samples of same depth pooled *in silico* are plotted in addition to individual ones.
(TIF)

Figure S2 **Cell density at different depths of Lake Arenguadi estimated using DAPI staining.** Grey squares indicate averages between counts and the solid lines 95% confidence intervals.
(EPS)

Figure S3 **Venn diagrams showing the distributions of shared OTUs within lakes.** Diagrams are annotated with the number of OTUs shared for each possible subset within (A) all amplicon datasets from a depths of 0 m in Lake Beseka; (B) all DNA amplicon datasets (excluding prefilter sample) and (C) all cDNA amplicon datasets across depths in Lake Beseka; (D) all DNA- and (E) all cDNA amplicon datasets in Lake Arenguadi between 0 to 3 meters.
(EPS)

Figure S4 **Average linkage clustering dendogram of amplicon sequence datasets using Bray-Curtis dissimilarity.**
(EPS)

Figure S5 **NMDS based on Bray Curtis dissimilarities between relative abundances of taxonomic groups.**
(EPS)

Figure S6 **Venn-diagrams illustrating partitioning of variation of selected physicochemical variables.** Partitioning of total community. As response variable, OTU abundance across individual sequence datasets was used in (A) and OTU abundance across habitats (pooled datasets) in (B).
(EPS)

Figure S7 **Bray-Curtis community dissimilarity between surface samples from different lakes plotted vs. the physical distance between lakes.** Where replicate surface samples existed, the average composition was used. Minimum distances between lakes were measured using Google Maps.
(PDF)

Table S1 **Full list of sampling sites and environmental parameters measured during sampling.**
(DOCX)

Table S2 **Overview of sequence datasets.**
(DOCX)

Table S3 **Taxonomic composition of all sequence datasets.**
(XLS)

Table S4 **UniRef90 protein clusters.** Accession, name and length of representative protein sequence for UniRef with number of putative mRNA reads sharing best alignments to each cluster, mean read length and coverage (total read length/protein sequence length in bp). Only clusters >2 mRNA reads and aligning with a bitscore >45 included.
(XLSX)
