# Supplementary figures and images for: Surprising Prokaryotic and Eukaryotic Diversity, Community Structure and Biogeography of Ethiopian Soda Lakes
Source: PLoS One. 2013 Aug 30;8(8):e72577. doi: 10.1371/journal.pone.0072577 (PMC3758324; doi:10.1371/journal.pone.0072577)

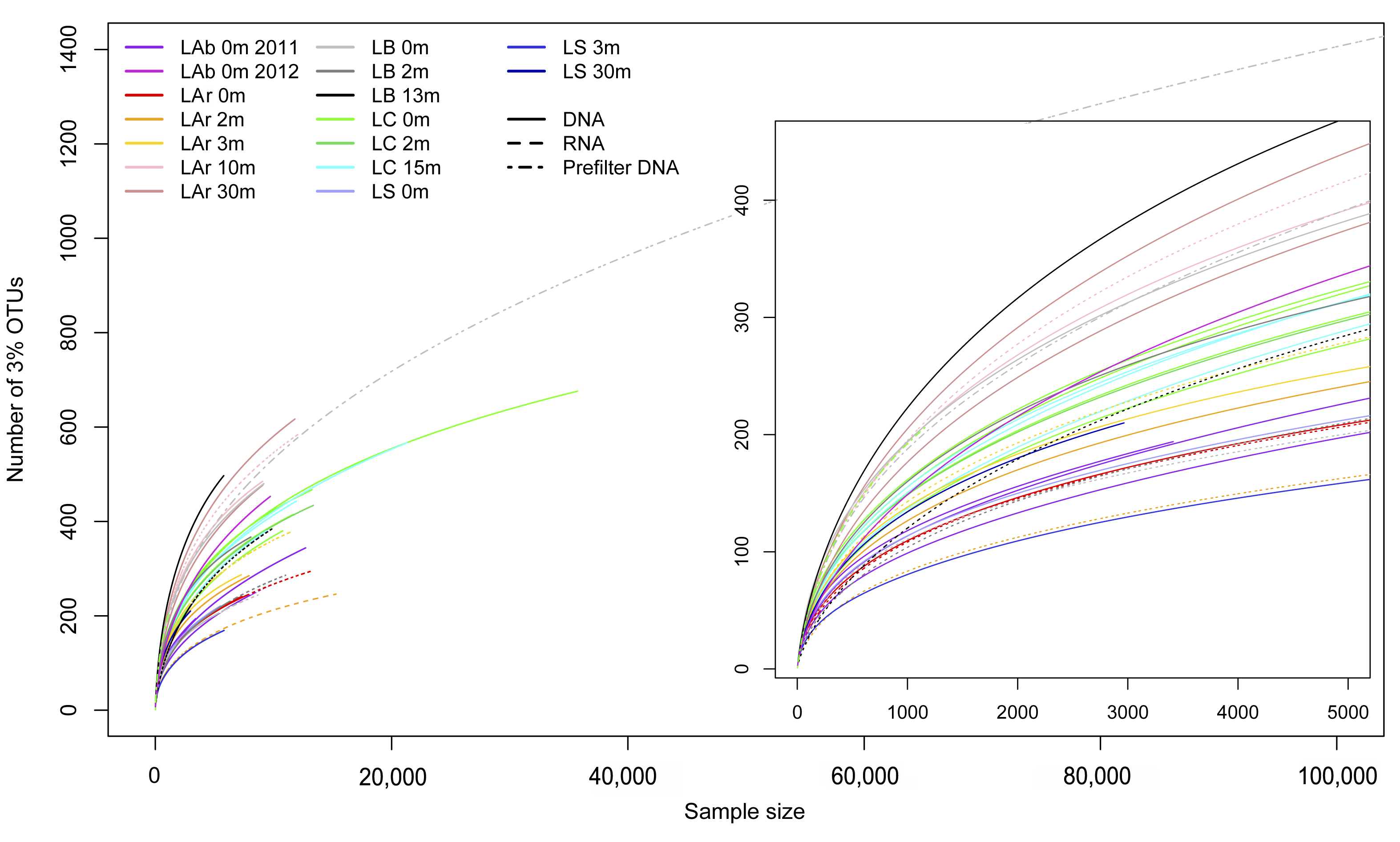

Supplement: Figure S1 — Rarefaction curves of OTUs from amplicon samples. The number of encountered OTUs (perceived richness) is plotted relative to sub-sampled sequence datasets size, i.e. number of reads. For Chitu and Abijata, samples of same depth pooled in silico are plotted in addition to individual ones. (TIF) [file pone.0072577.s001.tif]

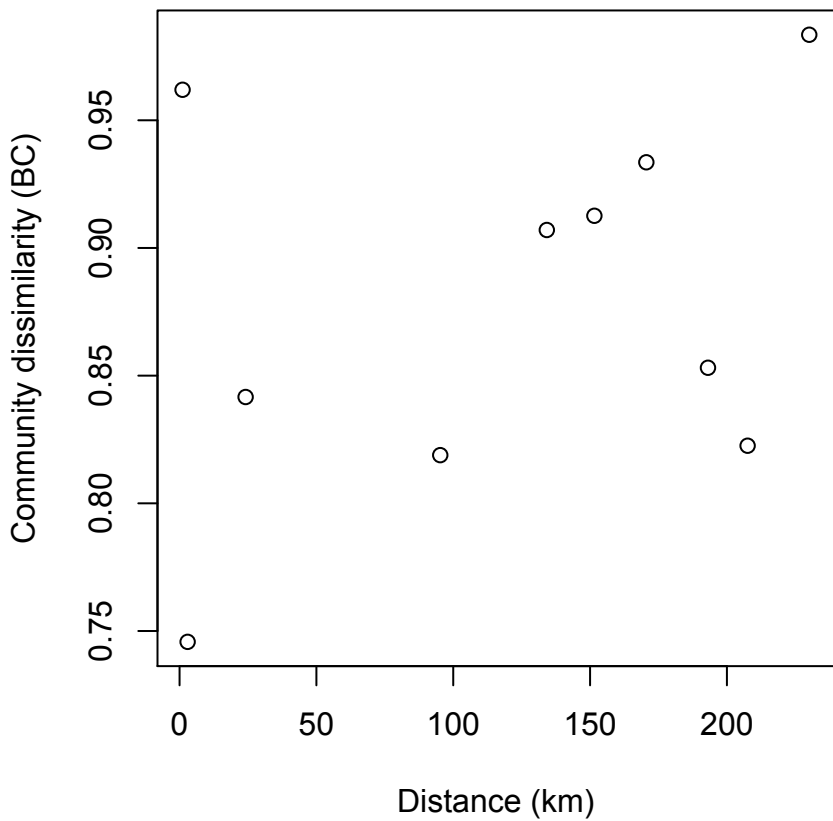

Supplement: Figure S7 — Bray-Curtis community dissimilarity between surface samples from different lakes plotted vs. the physical distance between lakes. Where replicate surface samples existed, the average composition was used. Minimum distances between lakes were measured using Google Maps. (PDF) [file pone.0072577.s007.pdf]
