# Supplementary material for: Surprising Prokaryotic and Eukaryotic Diversity, Community Structure and Biogeography of Ethiopian Soda Lakes
Source: PLoS One. 2013 Aug 30;8(8):e72577. doi: 10.1371/journal.pone.0072577 (PMC3758324; doi:10.1371/journal.pone.0072577)
Supplement: Table S1 — Full list of sampling sites and environmental parameters measured during sampling. (DOCX) [file pone.0072577.s008.docx]

**Supplementary table S1 –** Full list of samples and environmental parameters measured during sampling

| **Site name** | **Lake** | **Latitude (°N)** | **Longitude (°E)** | **Depth (m)** | **pH** | **Salinity** | **O_2_ (ppm)** | **Temp. (°C)** | **Sechi depth** | **Sampling date** | **Sample vol. (ml)** |
| --- | --- | --- | --- | --- | --- | --- | --- | --- | --- | --- | --- |
| LAb A | Abijata | 7.57655 | 38.63485 | 0 | 9.9 | 3.4% | ND | 21.5 | 0.55m | 30.3.2011 | 540 |
| LAb B | Abijata | 7.57583 | 38.63420 | 0 | 9.9 | 3.4% | ND | ND | ND | 30.3.2011 | 550 |
| LAb C | Abijata | 7.66364 | 38.59417 | 0 | 10.0 | 3.2% | ND | ND | ND | 26.12.2011 | 200 |
| LAr 0 | Arenguadi | 8.69483 | 38.97733 | 0 | 9.85 | 0.28% | 11.5 | 24 | 0.33m | 27.3.2011 | 250 |
| LAr 2 | Arenguadi | 8.69483 | 38.97733 | 2 | 9.85 | 0.27% | 10.0 | ND | - | 27.3.2011 | 250 |
| LAr 3 | Arenguadi | 8.69483 | 38.97733 | 3 | 9.87 | 0.27% | 10.0 | ND | - | 27.3.2011 | 240 |
| LAr 10 | Arenguadi | 8.69483 | 38.97733 | 10 | 9.72 | 0.21% | 0.0 | ND | - | 27.3.2011 | 500 |
| LAr 30 | Arenguadi | 8.69483 | 38.97733 | 30 | 9.65 | 0.26% | 0.0 | 20 | - | 27.3.2011 | 300 |
| LB 0 | Beseka | 8.90065 | 39.87483 | 0 | 9.65 | 0.29% | ND | 25.5 | ND | 31.3.2011 | 1300 |
| LB 2 | Beseka | 8.90065 | 39.87483 | 2 | 9.64 | 0.30% | ND | 25.0 | - | 31.3.2011 | 1400 |
| LB 13 | Beseka | 8.90065 | 39.87483 | 13 | 9.61 | 0.31% | ND | 24.5 | - | 31.3.2011 | 1200 |
| LCA0 | Chitu | 7.40490 | 38.42073 | 0 | 10.3 | 5.8% | 0.3 | 24 | 0.30m | 29.3.2011 | 600 |
| LCA2 | Chitu | 7.40490 | 38.42073 | 2 | 10.4 | 5.8% | 0.0 | ND | - | 29.3.2011 | 520 |
| LCA15 | Chitu | 7.40490 | 38.42073 | 15 | 10.4 | 5.8% | 0.0 | 22 | - | 29.3.2011 | 130 |
| LCB0 | Chitu | 7.40573 | 38.42180 | 0 | ND | ND | 0.3 | ND | 0.30m | 29.3.2011 | 540 |
| LCB15 | Chitu | 7.40573 | 38.42180 | 15 | ND | ND | 0.0 | ND | - | 29.3.2011 | 350 |
| LCC0 | Chitu | 7.40483 | 38.42373 | 0 | ND | ND | 0.0 | ND | 0.30m | 29.3.2011 | 550 |
| LS 0 | Shalla | 7.47867 | 38.63017 | 0 | 9.8 | 1.8% | ND | ND | ND | 28.3.2011 | 1500 |
| LS 3 | Shalla | 7.47867 | 38.63017 | 3 | 9.8 | ND | ND | ND | - | 28.3.2011 | 1250 |
| LS 30 | Shalla | 7.47867 | 38.63017 | 30 | ND | ND | ND | 22 | - | 28.3.2011 | 1600 |
