# Supplementary material for: Surprising Prokaryotic and Eukaryotic Diversity, Community Structure and Biogeography of Ethiopian Soda Lakes
Source: PLoS One. 2013 Aug 30;8(8):e72577. doi: 10.1371/journal.pone.0072577 (PMC3758324; doi:10.1371/journal.pone.0072577)
Supplement: Table S2 — Overview of sequence datasets. (DOCX) [file pone.0072577.s009.docx]

**Supplementary table S2 – Overview of sequence datasets**

| **Site name** | **Nucleic acid extracted** | **Filter type^a^** | **Nucleic acid conc. (ng/ml)** | **PCR cycles^b^** | **Raw reads** | **Filtered reads** | **Chimeric reads (%)** | **SSU rRNA reads^c^** | **mRNA reads^d^** | **OTUs** | ***H’* ^e^** | **(1-D) ^e^** |
| --- | --- | --- | --- | --- | --- | --- | --- | --- | --- | --- | --- | --- |
| LAb A | DNA | 0.2μm | 6 | 30 | 11319 | 9498 | 2.0% | 9331 | - | 260 | 3.4 | 0.92 |
| LAb B | DNA | 0.2μm | 9 | 30 | 3986 | 3443 | 0.9% | 3410 | - | 194 | 3.8 | 0.95 |
| LAb C | DNA | NF | 15 | 25 | 11769 | 9783 | 0.5% | 9733 | - | 453 | 3.7 | 0.93 |
| LAr 0 | RNA | 0.2μm | 41 | 0 | 163674 | 147610 | - | 49077 | 5405 | N/A | N/A | N/A |
| LAr 0 | DNA | 0.2μm | 12 | 22 | 8412 | 7340 | 0.4% | 7326 | - | 241 | 3.5 | 0.94 |
| LAr 0 | RNA | 0.2μm | 41 | 22 | 15512 | 13121 | 3.4% | 13099 | - | 294 | 3.4 | 0.92 |
| LAr 2 | DNA | 0.2μm | 15 | 22 | 9310 | 7933 | 0.2% | 7910 | - | 285 | 3.7 | 0.94 |
| LAr 2 | RNA | 0.2μm | 14 | 22 | 17892 | 15306 | 3.1% | 15281 | - | 246 | 2.3 | 0.68 |
| LAr 3 | DNA | 0.2μm | 7 | 22 | 8365 | 7279 | 0.4% | 7255 | - | 287 | 4.0 | 0.96 |
| LAr 3 | RNA | 0.2μm | 32 | 22 | 13321 | 11405 | 1.7% | 11381 | - | 377 | 3.9 | 0.95 |
| LAr 10 | DNA | 0.2μm | 29 | 22 | 11008 | 9086 | 0.5% | 9080 | - | 485 | 4.3 | 0.96 |
| LAr 10 | RNA | 0.2μm | 51 | 19 | 14807 | 12068 | 2.6% | 12051 | - | 584 | 4.2 | 0.94 |
| LAr 30 | DNA | 0.2μm | 6 | 22 | 11786 | 9104 | 0.3% | 9101 | - | 477 | 3.7 | 0.87 |
| LAr 30 | RNA | 0.2μm | 27 | 22 | 14870 | 11830 | 2.3% | 11815 | - | 617 | 4.3 | 0.94 |
| LB 0 | RNA | 0.2μm | 23 | 0 | 74630 | 67218 | - | 23051 | 1340 | N/A | N/A | N/A |
| LB 0 | DNA | 5μm | 55 | 25 | 137220 | 121642 | 0.5% | 121614 | - | 1519 | 4.1 | 0.39 |
| LB 0 | DNA | 0.2μm | 6 | 25 | 10902 | 9195 | 1.9% | 9176 | - | 480 | 4.7 | 0.98 |
| LB 0 | RNA | 0.2μm | 23 | 27 | 10837 | 8705 | 0.9% | 8691 | - | 244 | 3.3 | 0.90 |
| LB 2 | DNA | 0.2μm | 7 | 25 | 9403 | 8086 | 0.4% | 8079 | - | 367 | 4.0 | 0.94 |
| LB 2 | RNA | 0.2μm | 3 | 27 | 13324 | 11034 | 0.9% | 11018 | - | 286 | 3.2 | 0.90 |
| LB 13 | DNA | 0.2μm | 1 | 25 | 6884 | 5790 | 0.1% | 5782 | - | 498 | 4.6 | 0.97 |
| LB 13 | RNA | 0.2μm | 3 | 27 | 11401 | 9857 | 0.2% | 9854 | - | 384 | 2.6 | 0.73 |
| LCA0 | DNA | 5μm | 3 | 25 | 1319 | 1176 | 0.3% | 1172 | - | 207 | 3.9 | 0.91 |
| LCA0 | DNA | 0.2μm | 10 | 22 | 13454 | 11701 | 0.2% | 11689 | - | 415 | 4.2 | 0.97 |
| LCA2 | DNA | 0.2μm | 7 | 25 | 15562 | 13385 | 0.3% | 13351 | - | 434 | 4.1 | 0.97 |
| LCA15 | DNA | 0.2μm | 7 | 22 | 10822 | 9394 | 0.3% | 9382 | - | 380 | 3.9 | 0.95 |
| LCB0 | DNA | 0.2μm | 11 | 22 | 12447 | 10782 | 0.1% | 10763 | - | 380 | 3.8 | 0.95 |
| LCB15 | DNA | 0.2μm | 16 | 22 | 14147 | 11938 | 0.3% | 11930 | - | 443 | 4.1 | 0.96 |
| LCC0 | DNA | 0.2μm | 19 | 22 | 15560 | 13260 | 0.3% | 13246 | - | 467 | 4.3 | 0.97 |
| LS 0 | DNA | 0.2μm | N/A | 25 | 6326 | 5521 | 0.7% | 5509 | - | 221 | 3.7 | 0.94 |
| LS 3 | DNA | 0.2μm | N/A | 25 | 6698 | 5821 | 1.3% | 5817 | - | 169 | 3.1 | 0.91 |
| LS 30 | DNA | 0.2μm | N/A | 25 | 3329 | 2967 | 0.2% | 2967 | - | 210 | 3.8 | 0.95 |

^a^Type of filter from which nucleic acid was extracted: 0.2μm Sterivex, 5μm “pre-filter” or no filter (“NF”)

^b^ PCR cycles used for preparation of amplicon libraries. Zero values indicate shotgun sequencing libraries.

^c^Number of reads after noise-filtering and chimera removal with a minimum Megablast alignment bit-score of 155 to the SilvaMod database of small subunit (SSU) rRNA sequences

^d^Number of reads with a BLASTX alignment score above 45 to the UniRef90 protein sequence database.

^e^Shannon (*H’*) and Simpson (1-D) diversity indices estimated from the distribution of 3% OTUs for amplicon datasets.
